# Organization of the Proteostasis Network of Membraneless Organelles

**DOI:** 10.1002/advs.202500233

**Published:** 2025-06-11

**Authors:** Christine M. Lim, Yuqi Bian, Alicia González Díaz, Frank Pun, Alex Zhavoronkov, Richard I. Morimoto, Michele Vendruscolo

**Affiliations:** ^1^ Centre for Misfolding Diseases Yusuf Hamied Department of Chemistry University of Cambridge Cambridge CB2 1EW UK; ^2^ Insilico Medicine Hong Kong Science and Technology Park Unit 310, 3/F, Building 8W, Phase 2, Science Park Hong Kong China; ^3^ Department of Biochemistry Molecular Biology and Cell Biology Rice Institute for Biomedical Research Northwestern University Evanston IL 60208–3500 USA

**Keywords:** liquid‐liquid phase separation, membraneless organelles, protein condensation, protein homeostasis

## Abstract

Membraneless organelles (MLOs) are dynamic macromolecular condensates that act as crucibles to modulate cellular processes. Since MLOs form in the absence of lipid membranes, it is important to understand how their effective regulation is achieved by the protein homeostasis (proteostasis) system. To address this question, a comprehensive mapping of the proteostasis network (PN) of MLOs, comprising over 220 000 protein–protein interactions is reported. This analysis reveals how regulatory proteins (PN proteins) occupy central roles in the overall protein–protein interaction network of MLOs. It is then investigated which branches of the PN are most important in the regulation of MLOs, finding that the anabolic component, which makes up ≈30% of the PN, is more closely involved than the catabolic component, which makes up the remaining ≈70% of the PN. It is also found that translation‐related PN proteins and molecular chaperones play central roles in MLO regulation. Finally, how specificity may be achieved despite shared PN components is explored. These findings suggest that HSP70 chaperones function as generic MLO regulators, while client‐specific HSP70 co‐chaperones confer specificity to the chaperone action. These results identify the composition of the PN of MLOs, rationalize its organization, and reveal central roles of molecular chaperones in protein regulation within MLOs.

## Graphical abstract

A comprehensive mapping of the proteostasis network (PN) across membraneless organelles (MLOs) reveals how central PN components coordinate MLO regulation. The analysis uncovers principles of specificity and promiscuity within the PN. The findings suggest that HSP70 chaperones act as generic MLO regulators, while client specific HSP70 co‐chaperones may confer specificity to chaperone action.

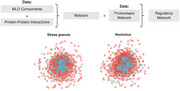

## 1 Introduction

Membraneless organelles (MLOs) are macromolecular assemblies that lack lipid membranes but are crucial for orchestrating key cellular processes such as gene regulation, RNA metabolism, and response to stress.^[^ ^1^, ^2^, ^3^, ^4^, ^5^ ^]^ Given their central roles in managing cellular homeostasis, the improper assembly and disassembly of MLOs may impair the optimal functioning of the cell, contributing to pathological phenomena.^[^ ^4^, ^5^, ^6^, ^7^ ^]^ For example, the dysregulated assembly of stress granules has been implicated in cancer, viral infection, and neurodegeneration, while the aberrant formation of nucleoli was found to occur in ageing and in diverse disease conditions.^[^ ^4^, ^5^, ^6^, ^7^ ^]^ Hence, the regulation of the macromolecular components of MLOs is essential for optimal cellular function.

In this context, the protein homeostasis (proteostasis) network (PN)^[^ ^8^, ^9^ ^]^ – the ensemble of proteins and protein–protein interactions involved in biological processes responsible for the expression and maintenance of the functional proteome^[^ ^10^, ^11^ ^]^ – is key in regulating the formation, clearance, composition, localization and physical properties of MLOs.^[^ ^12^, ^13^, ^14^, ^15^, ^16^ ^]^ Since MLOs are not bound by lipid membranes, and yet their mechanism of self‐assembly appears to be robust, one would like to understand how the different MLOs are specifically regulated to ensure that they reliably assemble and disassemble as they need to.

In an early study of the mechanisms of regulation of MLOs, morphological changes of nucleoli, Cajal bodies, splicing speckles, PML nuclear bodies, cytoplasmic processing bodies, and stress granules were monitored upon down‐regulation of 1354 genes.^[^ ^17^ ^]^ Based on that analysis, 80% of the targeted genes were found to modify the morphology of MLOs. However, ≈70% of the genes identified in that study affect multiple MLOs (Figure S1, Supporting Information), underscoring the complexity of the task of understanding the specific mechanisms at play in the regulation of individual MLOs by the PN.

To address this problem, we present a large‐scale mapping of the PN of MLOs and study its organization. We show that proteins in the PN (PN proteins) occupy central positions in the protein–protein interaction (PPI) networks of MLOs. By using the taxonomic structure recently proposed for the PN,^[^ ^8^, ^9^ ^]^ we then investigate which functional branches of the PN are most important in the regulation of MLOs, finding that a large proportion of translation‐related proteins and molecular chaperones are closely associated with MLOs. Next, we study the similarity between the PN regulation of different MLOs. A comparison of the PNs of different MLOs reveals that stress granules and P‐bodies share large portions of their individual PNs. Finally, we find that HSP70 proteins^[^ ^11^, ^18^ ^]^ are essential in modulating MLO disassembly, with HSP70 exerting promiscuous activity on MLO regulation while the co‐chaperones of HSP70 enable specificity. Overall, the mapping of the PN of MLOs that we report, together with its initial analysis, offers a tool for the investigation of the mechanism of regulation of these important cellular structures.

## 2 Results

### 2.1 Comparison and Benchmarking of Databases Reporting Protein‐MLO Localization

To map and study the PN of MLOs, we first obtained data on protein localization in various MLOs. For this purpose, we explored four databases reporting component identification of MLOs: i) DrLLPS (http://llps.biocuckoo.cn/),^[^ ^19^ ^]^ which reports a manual collection of proteins that participate in the phase separation of MLOs (categorized by MLO), annotated in 8 model organisms (including *Homo sapiens*), ii) PhaSepDB (http://db.phasep.pro/),^[^ ^20^, ^21^ ^]^ which, as of June 2022, contains protein component data of 73 MLOs including 770 low‐throughput entries and 7307 high throughput entries from multiple organisms, iii) CD‐CODE (https://cd‐code.org/),^[^ ^22^ ^]^ which is a crowdsourced database offering a community‐editable platform for reporting biomolecular condensate constituents, and iv) PhaSePro (https://phasepro.elte.hu),^[^ ^23^ ^]^ which is a manually‐curated database reporting proteins verified to drive phase separation in vivo and in vitro. It contains just over 100 proteins from multiple organisms, including eukaryotes, bacteria, and viruses.

To select a database for our study, we compared the data contents of *Homo sapiens* proteins. DrLLPS, PhaSepDB, CD‐CODE, and PhaSePro contain information on 3545, 4413, 3851, and 59 proteins, respectively (Section S1, Supporting Information). Since PhaSePro contained very few *Homo sapiens* proteins compared to the other databases, we omitted it from further analysis and proceeded to study the consistency in protein‐MLO localization across the other 3 databases (Figure S2, Supporting Information). We found 1835 proteins to be consistently reported across all DrLLPS, PhaSepDB, and CD‐CODE (Figure S2A, Supporting Information). Of the 3 databases, DrLLPS had the highest rate of corroboration with other datasets, since 98% (3490 of 3545) of the proteins within its database are reported in either PhaSepDB or CD‐CODE, or both. CD‐CODE had the next highest rate of corroboration with other datasets, with 94% (3618 of 3851) of the proteins within its database being reported in DrLLPS or PhaSepDB. PhaSepDB had the lowest rate of corroboration with other datasets, with 50% (2199 of 4413) of the proteins within its database being reported in DrLLPS or CD‐CODE. Since we intended to use the data for mapping the PN of MLOs, we further compared the reporting of PN protein localization in MLOs (Figure S2B, Supporting Information). Upon comparison, we found 575 PN proteins reported in all the 3 databases compared. Also in this case, DrLLPS had the highest rate of corroboration with other datasets, with 98% (906 of 922) of the PN proteins within its database being reported in at least 1 of the other 2 databases, followed by CD‐CODE, with 97% (942 of 972), and PhaSepDB, with 70% (649 of 917). We also compared the number of MLOs annotated within the 3 databases (Figure S2C, Supporting Information). 12 MLOs are annotated across all 3 databases: Nuclear Speckles, Nucleoli, Paraspeckles, Stress Granules, Sam68 Nuclear Bodies, Centrosomes, Cajal Bodies, PML Nuclear Bodies, Nuclear Pore Complexes, Histone Locus Bodies, Nuclear Stress Bodies. We note that Post‐Synaptic Densities (PSD) are annotated in both DrLLPS and CD‐CODE, but PSD components are reported together with components of presynaptic clusters in PhaSepDB.

We continued to study whether the reported proteins are annotated as components of the same MLO across datasets (Figure S3, Supporting Information). We carried out this analysis for 11 MLOs with the highest number of unique components across all 3 studied databases (n_components_ >100, Section S1, Supporting Information). Of the 11 MLOs compared, 7 show high consistency in the reported protein‐MLO localization (at least 100 proteins that were common to at least 2 datasets). The 7 MLOs with high consistencies (“best‐profiled”) are the: Nucleolus, Stress Granule, Post‐Synaptic Density, P‐body, Centrosome, Nuclear Speckle, and PML Nuclear Body. These 7 MLOs were hence used for the mapping of the PN of MLOs further in this study.

Overall, we selected DrLLPS for our study, since it had the highest rate of corroboration, in terms of proteins reported and protein‐MLO localization annotations. A high rate of corroboration acted as a proxy for confidence since the databases were largely manually attributing higher confidence from recognition by multiple reviewers of the cited evidence.

### 2.2 Mapping the Proteostasis Network of MLOs

**Figure 1:**
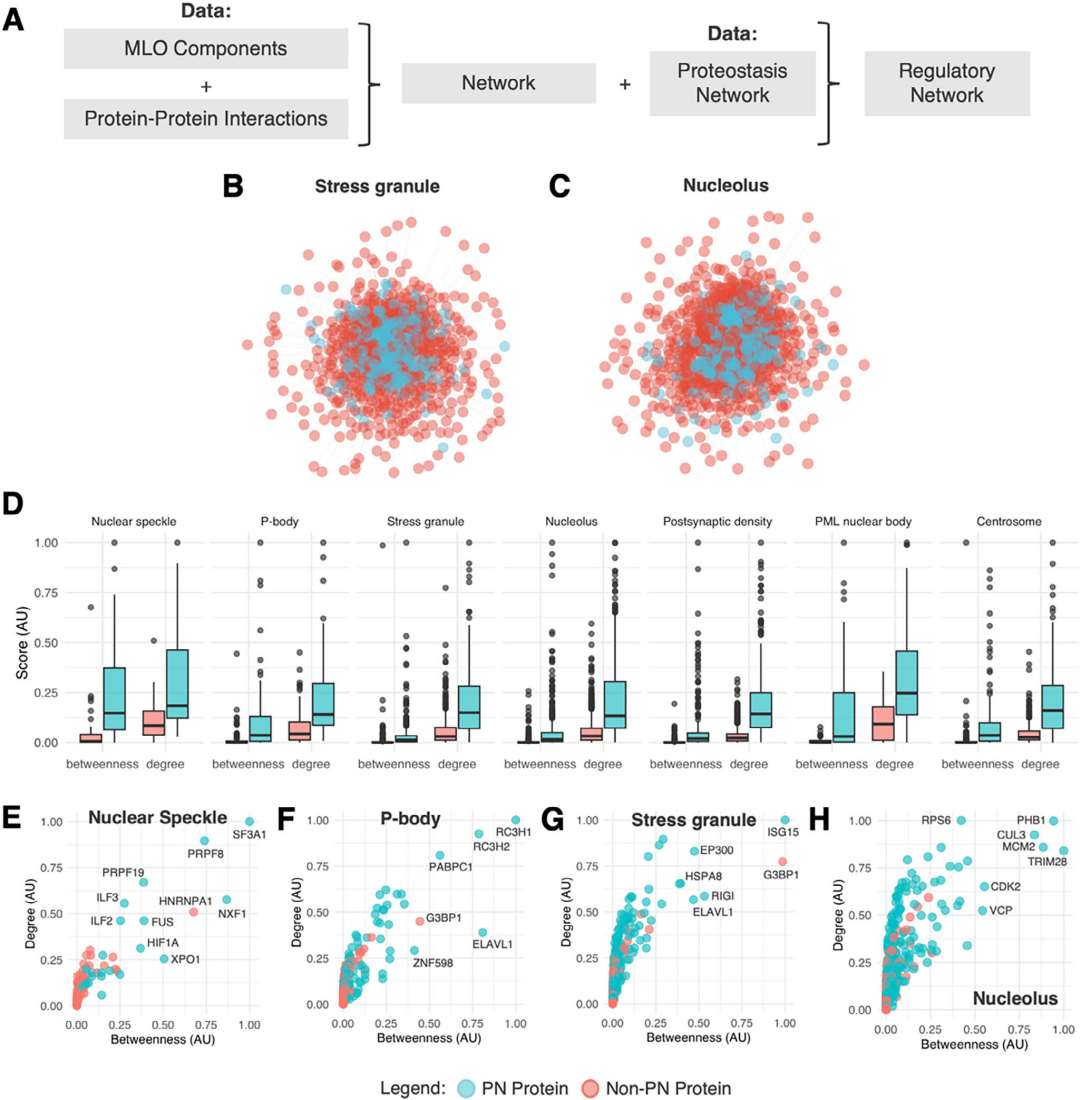
Mapping and centrality analysis of the proteostasis network (PN) of MLOs. A) Flowchart illustrating the procedure used to determine the MLO‐specific PNs. The protein components of a given MLO were obtained from DrLLPS,^[^ ^19^ ^]^ and the corresponding protein–protein interactions were extracted from BioGRID.^[^ ^24^ ^]^ The subset of protein–protein interactions involving PN components was then identified as the MLO‐specific PN, i.e., the regulatory network of an MLO. B,C) Schematic illustration of representative MLO‐specific PNs for stress granules (B) and nucleoli (C). Blue nodes in the protein–protein interaction network represent PN proteins, and red nodes represent non‐PN proteins. In these regulatory networks, PN proteins tend to be found in the center of the network, suggesting their essentiality within these MLO‐specific PNs. D) Centrality scores, including betweenness and degree, are calculated and compared for PN versus non‐PN proteins within each MLO‐specific PNs. PN proteins have a higher degree and betweenness compared to non‐PN proteins, indicating their importance in network regulation. E–H) PN proteins tend to be hubs and bottlenecks (hub: high degree; bottlenecks: high betweenness) of representative MLOs–Nuclear speckles (E) P‐bodies (F), stress granules (G), and nucleoli (H).

To comprehensively map the PN of MLOs, we generated the MLO‐PN network for each individual MLO before combining them into a comprehensive MLO‐PN map. The steps taken to generate the PN network of each individual MLO are illustrated in **Figure** **1**A. In brief, for each MLO, we first identified its components (using DrLLPS^[^ ^19^ ^]^ as selected earlier) and generated the protein–protein interaction networks between the MLO components and PN proteins according to the protein interaction data in BioGRID's^[^ ^24^ ^]^ (refer to Methods). Representative MLO‐PN networks for the 7 best‐characterized MLOs (Figure S3, Supporting Information)–stress granules, P‐bodies, nucleoli, PML nuclear bodies, centrosomes, and post‐synaptic densities are illustrated in the text/figures. The edge lists summarizing protein interactions for each MLO‐PN network are available in Section S2 (Supporting Information).

### 2.3 PN Proteins Occupy Central Positions in the PPI Networks of MLOs

To compare the regulatory roles of PN proteins against non‐PN proteins within the overall PPI network of MLOs. A protein's role in regulation of the MLO network was estimated using 2 network measures (see Experimental Section): 1) the degree, i.e., the number of connections of a protein within the network, and 2) the betweenness, i.e., the extent to which a protein lies on the shortest path between protein pairs within the network. These two measures are useful because hub proteins (high degree) and bottleneck proteins (high betweenness) were shown to play crucial topological and functional roles in maintaining the integrity and functionality of protein–protein interaction networks.^[^ ^25^ ^]^ Our analysis reveals that PN proteins tend to have higher degrees and higher betweenness within MLO PPI networks (Section S3, Supporting Information).

In these regulatory networks, PN proteins tend to be found in central positions in the network, as shown for representative MLO‐specific PN for stress granules (Figure 1B) and nucleoli (Figure 1C). A comparison of centrality values between PN and non‐PN proteins revealed that PN proteins are central in the MLOs included in this study (Figure 1D). A twin‐axis landscape of degree and betweenness is presented for representative MLOs: Nuclear speckles (Figure 1E), P‐bodies (Figure 1F), stress granules (Figure 1G), and nucleoli (Figure 1H). These results highlight that essential proteins, for example, G3BP1, which is a key scaffold of stress granules (Figure 1G), and HNRNPA1, which is a scaffold of nuclear speckles (Figure 1E), are hub‐bottlenecks. These proteins strongly disrupt the MLO network and its formation upon removal.^[^ ^26^ ^]^

### 2.4 Functional Branches of the PN of MLOs

**Figure 2:**
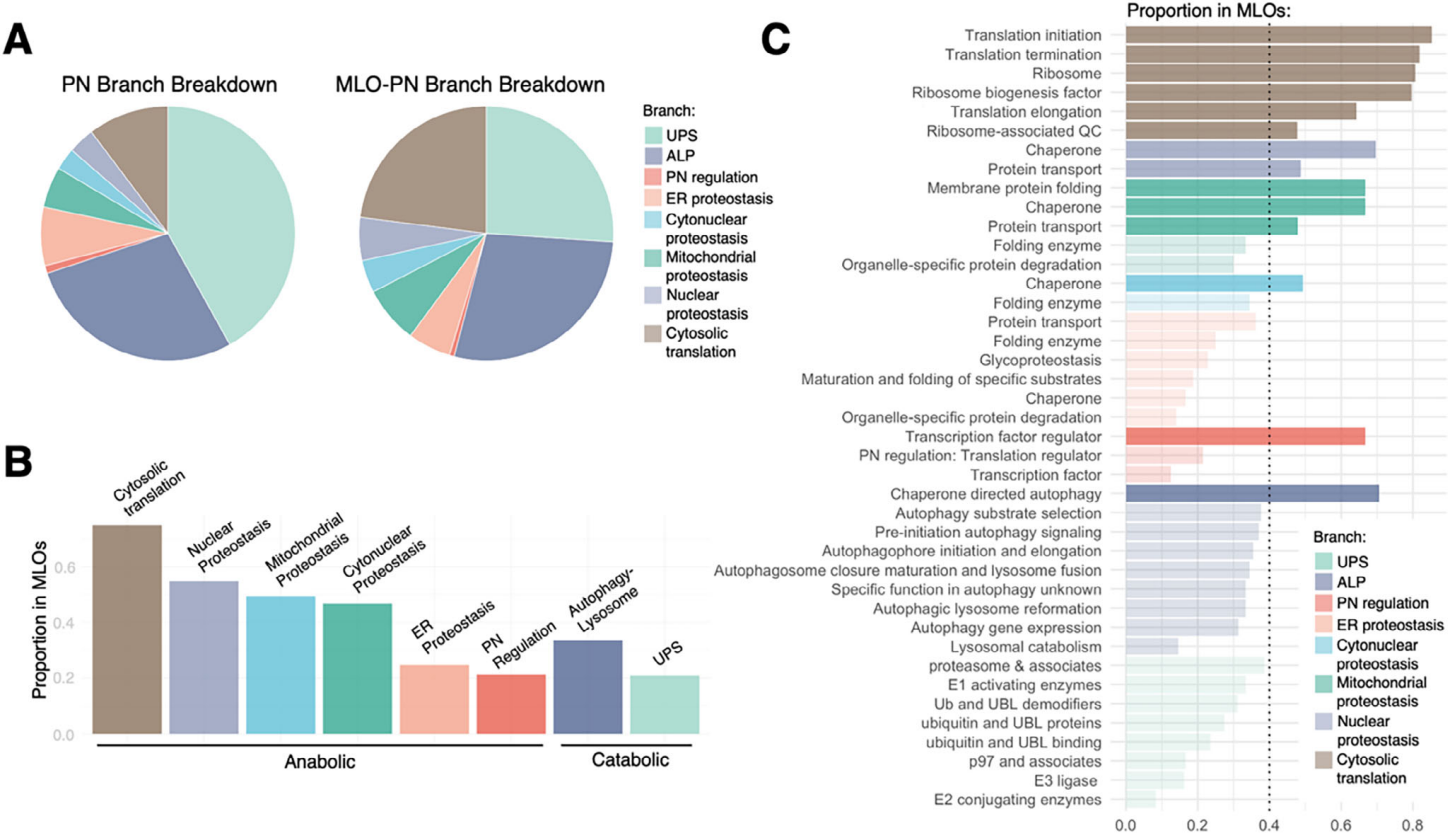
Analysis of the functional branches and functional classes of the PN of membraneless organelles. A) Comparison of the distribution of the various functional PN branches across the overall PN and the MLO‐specific PN. B) Proportion of each PN branch involved in membraneless organelles. A larger proportion of anabolic PN proteins are involved in membraneless organelles compared to catabolic PN proteins. C) Proportion of each PN class involved in membraneless organelles. A large proportion of translation‐related proteins are involved in membraneless organelles. In addition, molecular chaperones are also largely involved in membraneless organelles.

To study the organization of the PN of MLOs, we calculated the size of the different PN branches (PN branches) relative to the overall PN^[^ ^8^, ^9^ ^]^ by dividing the number of proteins in each PN branch by the total number of proteins in the PN. We then calculated the size of the different PN branches relative to the PN of MLOs by dividing the number of proteins in MLOs in each PN branch by the total number of proteins in the PN of MLOs. We found that the regulation of protein synthesis in the cytosol (“Cytosolic translation”) is predominantly carried out by the PN of MLOs (**Figure** **2**A). Similarly, the regulation at the subcellular level (“Mitochondrial proteostasis”, “Nuclear proteostasis,” and Cytonuclear proteostasis’) is also predominantly carried out by the PN of MLOs (Figure 2A). By contrast, the regulation of protein degradation (“UPS”) involves only a limited manner in the PN of MLOs (Figure 2A).

To support these conclusions, for each PN branch, we calculated the number of proteins in the PN of MLOs divided by a number of proteins in the PN (Figure 2B). We found that ≈80% of the regulation of protein synthesis in the cytosol (“Cytosolic translation”) is associated with MLOs (Figure 2B). Similarly, we found that ≈50% of the subcellular proteostasis (“Mitochondrial proteostasis”, “Nuclear proteostasis,” and Cytonuclear proteostasis’) is associated with MLOs (Figure 2B). By contrast, we found that the catabolic proteostasis (“Autophagy‐Lysosome Pathway” and “UPS”), which makes up 70% of the overall PN, is not prevalently associated with MLOs (Figure 2B).

### 2.5 Functional Classes of the PN of MLOs

To continue the study of the organization of the PN of MLOs, we analyzed the PN classes, which are the level below the PN branch in the taxonomy scheme of the PN.^[^ ^8^, ^9^ ^]^ For each PN class, we calculated the number of proteins in that class in the PN of membraneless organelles divided by a number of proteins in that class in the PN (Figure 2C). We found that several PN classes involved in protein synthesis (“Transcription factor regulator”, “Translation initiation”, “Translation termination”, “Translation elongation, ‘Ribosome”, and “Ribosome‐associated QC”) are closely associated with MLOs.

### 2.6 Similarity Between the PN of MLOs

**Figure 3:**
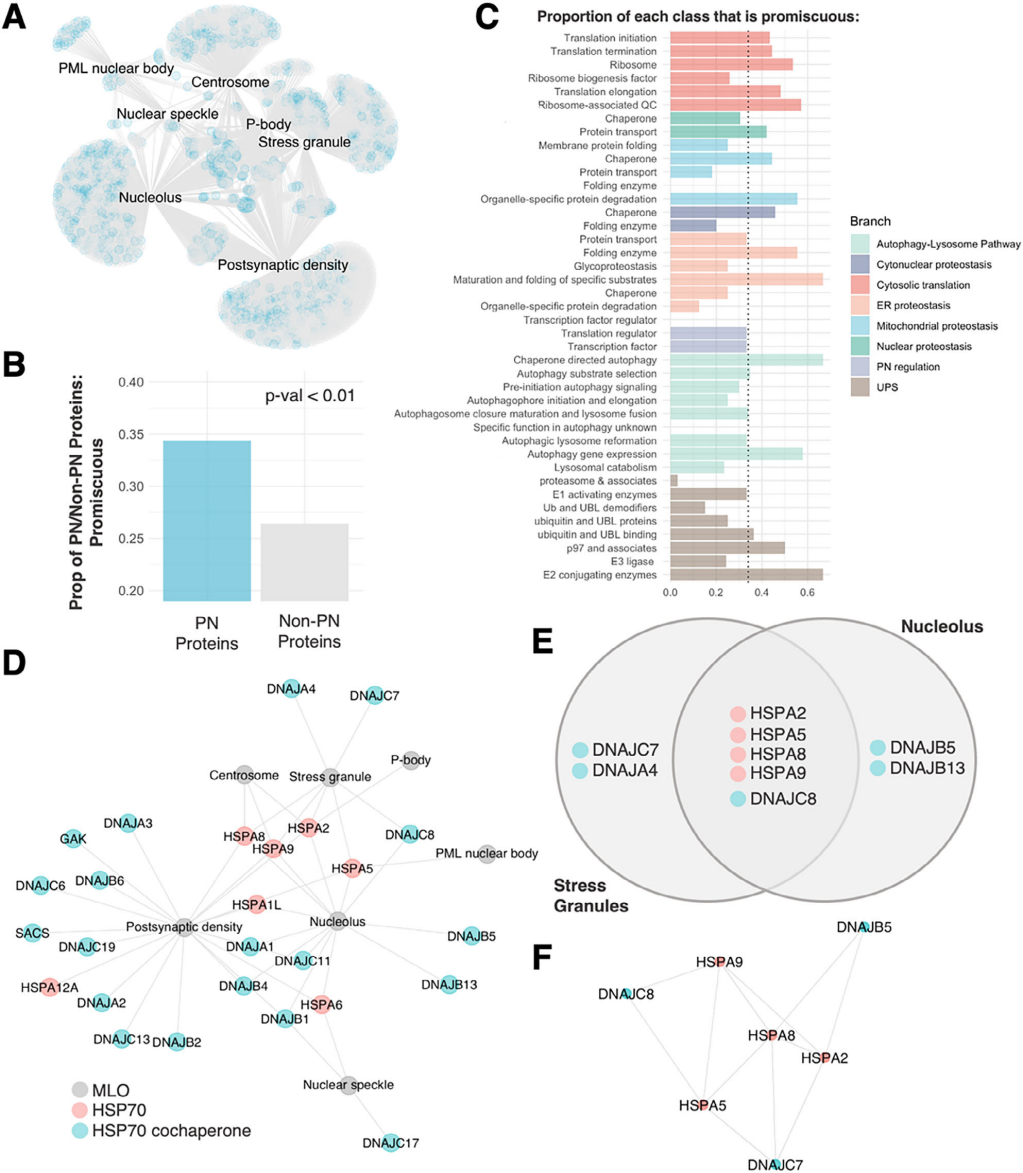
Similarity of MLO‐PN components. A) Overall organization of the PN that regulates MLOs (PML nuclear bodies, centrosomes, stress granules, P‐bodies, nucleoli, and post‐synaptic densities). Blue nodes in the protein–protein interaction network represent PN proteins, and gray nodes represent non‐PN proteins. Many PN proteins are involved in multiple MLOs. The full list of proteins and interactions is provided in Section S2 (Supporting Information). B) Proportion of PN or Non‐PN proteins within the MLO‐PN networks that are promiscuous. Statistical significance was determined using the χ^2^ test. C) To determine the MLO‐specificity of the PN classes, we compute the proportion of each PN class that was found to localize in >1 MLO (promiscuous). The dotted line represents the reference level–proportion of PN proteins (general) that are promiscuous. D) HSP70 chaperones (pink) tend to be present in multiple MLOs, while HSP70 co‐chaperones (teal) tend to be more specific in their MLO localization. E) HSPA2, HSPA5, HSPA8, and HSPA9 are HSP70 chaperones present in both stress granules and nucleoli. In contrast, while DNAJC8 is common to both MLOs, DNAJC7 and DNAJA4 localize in stress granules, while DNAJB5 and DNAJB13 are localizing in nucleoli. F) Interaction network of HSP70 proteins common to stress granules and nucleoli and selected co‐chaperones.

When the individual MLO‐PN networks are pulled together, the overall map of MLO‐PN networks (**Figure** **3**A) reveals that many PN proteins are shared among multiple MLOs. Notably, a larger percentage of PN proteins are promiscuous to multiple MLOs as compared to non‐PN proteins (Figure 3B).

Seeking to understand if specific PN functional classes are involved in promiscuous activity, we calculated the proportion of proteins found in one or more MLOs per PN class (Figure 3C). We found that a larger proportion of proteins involved in cytosolic translation, chaperones, autophagy gene translation, and E2 ligases tended to be promiscuous relative to the general level of PN promiscuity across MLOs computed in Figure 3B. While most PN proteins tend to be involved in only one or two MLOs, some PN classes, such as chaperones, are involved in as many as 5 MLOs. This is in line with known information in the literature, whereby molecular chaperones are known to be promiscuous to a variety of clients, enabling the cell to deal with protein misfolding and aggregation, with their client selectivity modulated via their co‐chaperones. We find that several molecular chaperones tend to be more promiscuous than others, with HSPA2 localizing in 5 MLOs, and HSPA5/HSPA8/HSPA9 localizing in 4 MLOs. This raised a question on how the specificity of MLO regulation is enabled, given that PN proteins, such as chaperones, co‐localize to multiple MLOs and that MLOs share a large proportion of PN proteins. For this purpose, we compared 2 families of proteins known to exhibit client substrate specificity – molecular chaperones and E1, E2, and E3 ligases. We found that several molecular chaperones (≈40 out of 220) are shared across multiple MLOs (Figure S4, Supporting Information) while E1, E2, and E3 ligases retain the trend of substrate‐specificity by being more MLO‐specific, with few ligases common to multiple MLOs (Figure S4, Supporting Information). Hence, we proceed to further clarify how molecular chaperones, which were shown earlier to be closely associated with MLOs, enable substrate specificity.

To further illustrate how molecular chaperones may enable specificity in MLOs regulation, we studied the HSP70 systems,^[^ ^11^, ^18^ ^]^ which assist the folding and binding of client proteins. The functional properties of the HSP70 systems are critically dependent upon HSP70 co‐chaperones to bind and execute their function on substrate proteins. We hence hypothesized that HSP70 chaperones may have broad regulatory functions on multiple MLOs, while co‐chaperones may have MLO‐specific regulatory capabilities. To test this hypothesis, we first mapped out the localization of HSP70 chaperones and their co‐chaperones. Our localization map revealed that HSP70 proteins are indeed promiscuous to multiple MLOs, while HSP70 co‐chaperones tend to localize to different types of MLOs (Figure 3D). Analyzing stress granules and nucleoli as case studies, we found that they share four HSP70 chaperones (HSPA2, HSPA5, HSPA8, and HSPA9), but only share one HSP70 co‐chaperone (DNAJC8, Figure 3E). Interactions between the shared HSP70 chaperones and three types of HSP70 co‐chaperones (SG‐specific, nucleoli‐specific, and shared) are presented (Figure 3F).

### 2.7 HSP70s are Generic, While HSP70 Co‐Chaperones Enable Specificity in the Regulation of MLOs

For experimental validation, we selected the following proteins: i) HSPA2 and HSPA5, which are HSP70 chaperones common to both stress granules and nucleoli, ii) DNAJC7, which is a SG‐specific HSP70 co‐chaperone, iii) DNAJB5, which is a nucleolus‐specific HSP70 co‐chaperone, and iv) DNAJA2, a HSP70 co‐chaperone taken as a negative control as it localizes to post‐synaptic densities within the MLO‐specific PN. We first checked the literature to ensure that these proteins were indeed found experimentally to be enriched in the relevant MLOs before proceeding to our experimental assay–HSP70^[^ ^27^ ^]^ and DNAJC7^[^ ^28^ ^]^ localization in stress granules upon sodium arsenite stress; HSP70^[^ ^29^, ^30^ ^]^ and DNAJB5^[^ ^31^ ^]^ localization in nucleoli. Within our experimental assay, we individually knocked down HSPA2, HSPA5, DNAJC7, DNAJB5, and DNAJA2 in SH‐SY5Y cells by incubating the cells with the corresponding siRNA for 72 h. We then studied the changes in stress granules and nucleoli observed upon triggering them via treating the cells with 0.5 mM sodium arsenite for 30 min or heating the cells at 42 °C for 30 min. Sodium arsenite is frequently used to trigger the formation of stress granules in various cell lines with incubation times up to 1 h,^[^ ^32^, ^33^ ^]^ while heat shock at ≈45 °C for 30 min has been shown to alter nucleoli morphology and increase HSP70 recruitment to the nucleoli in O23 hamster fibroblasts.^[^ ^34^ ^]^ Based on our hypothesis, we expected to see changes in both stress granules and nucleoli upon HSP70 protein knockdown, but MLO‐specific changes upon knockdown of HSP70 co‐chaperones, with the exception of the negative control DNAJA2.

**Figure 4:**
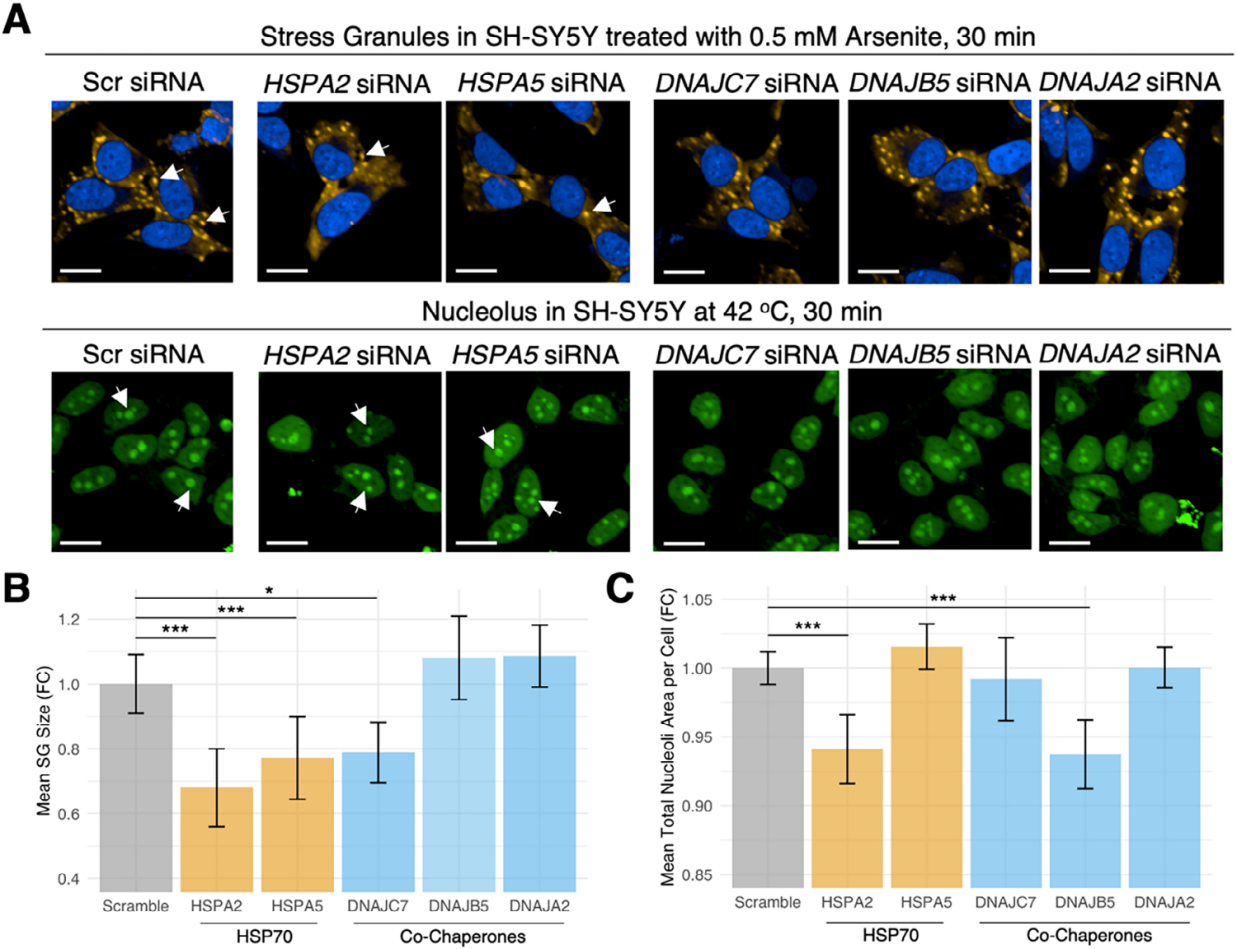
HSP70 proteins regulate multiple MLOs, while their co‐chaperones are specific to individual MLOs. A) Representative images of stress granules formed when SH‐SY5Y cells are treated with 0.5 mM of sodium arsenite for 30 min, and nucleoli observed upon heat stress at 42 °C for 30 min. B) Upon treatment with *HSPA2* siRNA, we observed a decrease in the average stress granule and nucleoli areas. Similarly, upon treatment with *HSPA5* siRNA, the average stress granule area decreased significantly. C) Average stress granule area only changes (decreases) upon treatment with *DNAJC7* siRNA and not *DNAJB5* or *DNAJA2* siRNA. Average nucleoli area only changes (decreases) upon treatment with *DNAJB5* siRNA and not *DNAJC7* or *DNAJA2* siRNA. N = 3 biological replicates of n = 6 technical replicates (for stress granules), and n = 3 technical replicates (for nucleoli). Statistical significance was determined using the Wilcoxon test. ^***^ *p*‐value< 0.05.

Our experimental results are consistent with our hypothesis that HSP70 chaperones are promiscuous, while HSP70 co‐chaperones may be specific to MLO regulation. To quantify this effect, we measured the average total area (size) of stress granules/nucleoli as a collective indicator of MLO presence, while noting that this measure does not necessarily correlate with the number, composition, or functionality of stress granules/nucleoli. Therefore, we interpret this as a preliminary indicator rather than a definitive readout of MLO regulation. This is because there is no clear indication whether fewer but larger stress granules/nucleoli or many but smaller stress granules/nucleoli are indicative of altered MLO assembly, disassembly, and/or dynamics. Based on our quantification, treatment with *HSPA2* siRNA decreased the average total area of stress granules and nucleoli upon 0.5 mM sodium arsenite and heat shock treatment, respectively (**Figure** **4**D), confirming the promiscuous activity of HSP70 chaperones across MLOs. Similarly, treatment with *HSPA5* siRNA also resulted in decreased total area of stress granules formed within each cell (Figure 4D,E). It is worth noting that HSPA2 transcript levels were found to be low under basal conditions in our system, requiring pre‐amplification for qPCR detection. This suggests limited expression under non‐stressed conditions, and we cannot rule out that its potential MLO regulatory function is activated primarily under cellular stress.

Our results from our knockdown experiment yield results that point in a similar direction to previous experiments–where treatment of sodium arsenite resulted in increased levels of HSP70 in rats,^[^ ^35^ ^]^ and increased HSP70 levels were shown to increase stress granule formation upon stress in YAMC cells.^[^ ^36^ ^]^ Hence, in the context of our experiment, knockdown of HSP70 transcripts likely prevented the increase in HSP70 usually triggered by sodium arsenite, and we see fewer (in terms of average total area) stress granules formed. This, however, contradicts other studies that show treatment with HSP70 siRNA does not affect stress granule formation (only disassembly) upon treatment with much lower concentrations of sodium arsenite.^[^ ^27^ ^]^ Other experiments have also shown that mutant HSP70 lacking chaperone activity was unable to localize to the nucleolus upon heat shock, coinciding with the non‐observation of swollen nucleoli usually observed upon heat shock.^[^ ^34^ ^]^ In contrast to the HSP70 promiscuity, treatment with siRNAs targeting HSP70 co‐chaperones resulted in higher specificity in targeting MLO formation. Treatment with *DNAJC7* siRNA resulted in a lowering of the total area of stress granules formed, but not the total area of nucleoli formed. In addition, treatment with *DNAJB5* siRNA resulted in a lower total area of nucleoli formed, but not the total area of the stress granules formed. Finally, treatment with our negative control *DNAJA2* siRNA did not affect the total area of stress granules or nucleoli formed within SH‐SY5Y cells (Figure 4D,F).

## 3 Discussion and Conclusion

In this work, we sought to understand the organization of the proteostasis system of MLOs by asking how different MLOs achieve functional specificity in protein quality control despite an overlap in their components. To address this question, we mapped the PN of MLOs and analyzed its organization. We thus identified the major components of this PN and measured the similarity of different MLOs in terms of their proteostasis regulation, finding several shared mechanisms.

To understand how the proteostasis system achieves regulation of MLOs despite these similarities, we explored how the PN components are capable of specifically modulating different MLOs. We showed how HSP70 chaperones are promiscuous in their regulatory activity across MLOs, while the co‐chaperones of HSP70 enable specificity in targeting MLOs. We note that our study points out the general role of HSP70s and their co‐chaperones in conferring specificity in MLO regulation in well‐characterized MLOs. Given that there are many other MLOs for which HSP70s or their co‐chaperones may not be detected, further work will be required to investigate the deterministic factors controlling the specificity of their modulation. Nevertheless, taken collectively, our results suggest that one of the key functions of molecular chaperones, in addition to regulating the conformational behavior of proteins during folding and binding, misfolding, and aggregation, is also to enable specificity in MLO regulation.

We acknowledge several limitations in the current study. First, our knockdown strategy was validated only at the mRNA level via qPCR. For proteins such as HSP70 chaperones, mRNA levels may not reflect actual protein abundance or activity. This caveat is particularly relevant for HSPA2, which exhibited low basal transcript levels in our SH‐SY5Y cultures. Further experiments will be needed to confirm protein‐level depletion and contextual expression dynamics of all explored targets in this paper. Second, we relied on changes in total MLO area as a morphological proxy for regulatory effects. However, this measurement does not capture the dynamics, properties, or functionality of MLOs (e.g., translational repression in stress granules, or rRNA synthesis in the nucleoli). Additional techniques will be needed to determine whether the observed morphological changes correspond to altered MLO characteristics and activity. Moreover, we recognize the limitations in drawing mechanistic conclusions from correlations. While the observed changes in MLO area upon knockdown are consistent with the hypothesis that HSP70 co‐chaperones may confer MLO‐specific regulation properties, direct demonstration of interaction (e.g., via co‐immunoprecipitation, proximity labelling, or live‐cell colocalization imaging) will be necessary to fully confirm this model. Finally, while our knockdown data support MLO‐specific effects, rescue experiments will be needed to definitively attribute phenotypes to specific protein depletion and rule out broader cellular stress responses to RNA interference.

The comprehensive mapping that we provided of the proteostasis system of MLOs will enable future investigations of several important aspects of the behavior of MLOs: 1) *Formation mechanisms*. Increasing evidence suggests that MLOs are formed by a phase separation mechanism.^[^ ^3^, ^4^ ^]^ It is still unclear, however, what the exact molecular and biophysical determinants are that drive the phase separation process leading to the formation of distinct MLOs; 2) *Response to environmental changes*. It will also be important to achieve a better understanding of how the assembly and disassembly of MLOs are dynamically regulated during various cellular conditions, such as stress or changes in metabolism.^[^ ^12^ ^]^ Understanding the triggers and mechanisms underlying these transitions would provide insights into the adaptability of the PN; 3) *Cross‐talk and coordination*. There is a need to reveal the nature of the cross‐talk between different MLOs and the mechanisms by which they coordinate their roles within the PN; 4) *Role of RNA in the self‐assembly and function*: RNA is a critical component in many MLOs, as RNA molecules can influence the dynamics, composition, and function of MLOs.^[^ ^37^, ^38^ ^]^ Detailed investigations of how RNA interacts with proteins within the PN to regulate the properties and functions of MLOs will provide a more comprehensive view of their regulation; and 5) *Pathological aggregation*. It remains to be clarified how the dysregulation of the self‐assembly process of MLOs leads to the pathological aggregation of proteins associated with neurodegenerative diseases, such as Alzheimer's disease and amyotrophic lateral sclerosis (ALS),^[^ ^4^, ^6^, ^7^ ^]^ and how the PN may help prevent this phenomenon.^[^ ^28^, ^39^ ^]^

We anticipate that further studies based on the mapping of the proteostasis system of MLOs will uncover more detailed mechanisms of the proteostasis regulation of these cellular structures, thus offering new avenues for target identification for therapeutic interventions for diseases associated with MLOs.

## 4 Experimental Section

### Proteostasis Network (PN) Data

A list of annotated PN components^[^ ^8^, ^9^ ^]^ was obtained from the Proteostasis Consortium (https://www.proteostasisconsortium.com/). This was used as the reference set of PN proteins in this study.

### MLO Component Data

Data on proteins reported to localize in MLOs from 4 databases were downloaded and compared to select the most suitable database for the study. The 4 databases are: DrLLPS^[^ ^19^ ^]^ (accessed Dec2023), PhaSepDB (version 2),^[^ ^20^, ^21^ ^]^ CD‐CODE (version 1.08),^[^ ^22^ ^]^ and PhaSePro.^[^ ^23^ ^]^ For all databases, only data entries for Species/Organism = “*Homo sapiens*” were included in this study. For DrLLPS, general entries (“Droplet” and “Others”) were removed from the dataset. The names of the MLOs annotated by the databases were standardized for quantification (Section S1, Supporting Information).

### Protein–Protein Interaction (PPI) Data

*Homo sapiens* PPI data were downloaded from BioGRID^[^ ^24^ ^]^ (accession date: Nov2023). Paired interactions from BioGRID were filtered for protein pairs that physically coexist (here‐after referred to as “physical‐PPI pairs”) using tags: “MI:0407 (direct interaction)”, “MI:0915 (physical interaction)”, “MI:0914 (association)”, and “MI:0403 (colocalization)”.

### Selection of Key MLOs for Study

MLO‐PN networks were hence generated for these 7 MLOs:Nucleolus, Stress Granule, Post Synaptic Density (PSD), P‐body, Centrosome, Nuclear Speckle, and PML Nuclear Body. These 7 MLOs were selected as they were determined to be the best‐profiled MLOs as per the number of unique protein components across DrLLPS, PhaSepDB, and CD‐CODE, n_components_ >100 (Section S1, Supporting Information), and high consistency in the reported protein‐MLO localization (Figure S3, Supporting Information).

### Setting up the MLO‐Specific PN‐Networks & Calculation of Centrality Scores

For each MLO included in the study, an MLO‐specific PN network was set up by integrating DrLLPS^[^ ^19^ ^]^ and the PPI data (obtained from BioGrid as described earlier). An MLO‐specific PPI edge list was generated by filtering the BioGrid interaction data for pairs that involved a) scaffold ↔ MLO component protein, or b) PN protein ↔ scaffold/MLO component protein. All self‐interactions were omitted from the interaction network. The undirected edge list for each MLO‐PN network is available in Section S2 (Supporting Information). Two centrality scores were calculated and used as a proxy for a protein's importance in regulating each MLO‐PN network. The centrality scores are: 1) the degree, i.e., the number of connections of a protein within the network, and 2) the betweenness, i.e., the extent to which a protein lies on the shortest path between protein pairs within the network. These were calculated for each protein in an MLO's MLO‐PN network using the betweenness() or degree() function within the igraph package in R.

### MLO PN Proteins Versus Non‐PN Proteins

Proteins within the PN network of each MLO were divided into 2 groups: PN proteins and non‐PN proteins. PN proteins within the protein network of an MLO are PN components that are part of the PN network of the MLO. Non‐PN proteins are the complementary set of proteins to PN proteins within the PN network of an MLO.

### Identification of Hub Proteins and Bottleneck Proteins in MLOs

To identify essential proteins in the MLO‐specific PNs using quantifiable metrics, the relative “hubness” and “bottleneckness” of individual PN components were determined within the MLO‐specific PNs. Hub proteins are proteins with a high degree within the network, and protein bottlenecks are proteins with high betweenness.^[^ ^26^ ^]^ The degree centrality (degree) of a node in a network refers to the number of its edges.^[^ ^40^ ^]^ Hence, in the analysis of MLO‐specific PNs, the degree of a protein refers to the number of neighboring proteins it interacts with. The betweenness centrality (betweenness) of a node quantifies the number of shortest paths passing through the node.^[^ ^37^, ^41^ ^]^ Nodes with high betweenness are critical points within a network, determining the amount of information flow, and are defined as bottleneck proteins.^[^ ^26^ ^]^ Bottleneck proteins are essential proteins, as failure of bottleneck proteins results in the collapse of the biological network. Hub‐bottlenecks, hence, refer to proteins with both a high degree and betweenness.^[^ ^26^ ^]^ Centrality scores are available in Section S3 (Supporting Information).

### Cell Culture Experiment

Human neuroblastoma cells (SH‐SY5Y) were cultured in DMEM/F‐12 GlutaMAX supplemented with 10% heat‐inactivated fetal bovine serum (hiFBS) and maintained at 37 °C, 5% CO_2_, 95% relative humidity. For each experiment, SH‐SY5Y cells were plated in PerkinElmer Cell Carrier Ultra plates at a density of 7.5k–10k cells/well. The cells were incubated for 24 h to allow adequate cell attachment before siRNA feeding. During siRNA feeding, full culture medium was replaced with fresh DMEM/F‐12 GlutaMAX with 1% hiFBS and fed with a final concentration of 10 nM siRNA. Lipofectamine RNAiMAX was used as the transfection reagent. The cells were incubated with Thermo Fisher Silencer Select siRNAs (HSPA2: #s20124 & #s20122, HSPA5: #s6979 & #s6980, DNAJC7: #s270217, DNAJB5: #s24554 & #s24556, and DNAJA2: #s6973 & #s7975) for 72 h before treatment with sodium arsenite or heat shock. The cells were treated with sodium arsenite or heat shock after 72 h, as optimal knockdown of the mRNA was reported to be attained usually 48–72 h after transfection.^[^ ^42^ ^]^ siRNA knockdown efficiency for all genes was confirmed via qPCR (Figure S5, Supporting Information). For sodium arsenite treatment, cells were supplied with fresh DMEM/F‐12 GlutaMAX, 1% hiFBS with 0.5 mM sodium arsenite and left to incubate for 30 min at 37 °C, 5% CO_2_, 95% relative humidity before fixing. For heat shock treatment, cells were incubated on a pre‐heated hot plate at 42 °C for 30 min before fixing. The SH‐SY5Y cells were fixed using 4% paraformaldehyde (PFA) dissolved in D‐PBS (+/+) and stored at 4 °C for less than a week before staining.

### RNA Extraction and Reverse Transcription

Total RNA was extracted using PureLink RNA Mini Kit (#12183018, Invitrogen) according to the manufacturer's protocol. 700 ng to 1 µg of RNA was reverse transcribed into cDNA in a 20 µL volume using the High‐Capacity cDNA Reverse Transcription Kit (#4368814, Applied Biosystems) according to the manufacturer's protocol.

### Quantitative PCR

The qRT‐PCR (*HSPA5*, *DNAJC7*, *DNAJB5*, and *DNAJA2*) was carried out in a volume of 20µl, which contained 1µl cDNA, 10µl TaqMan Gene Expression Master Mix (#4369016, Applied Biosystems), and 1x of each Taqman probe (ThermoFisher). The following reaction conditions were employed according to the manufacturer's instructions: 50 °C for 2 min, 95 °C for 10 min, then 40 cycles of 95 °C for 15 sec and 60 °C for 1 min. The relative level of mRNA was evaluated with the ΔΔCt method, using *GAPDH* as an endogenous control.

A preamplification reaction was carried out for *HSPA2* in a volume of 20 µl, which contained 4 µl cDNA, 10 µl TaqMan Gene Expression Master Mix (#4369016, Applied Biosystems), and 0.05x of each Taqman probes (ThermoFisher). The following reaction conditions were employed according to the manufacturer's instructions: 95 °C for 10 min, then 10 cycles of 95 °C for 15 sec and 60 °C for 4 min. The qRT‐PCR (HSPA2) was then carried out in a volume of 20 µl, which contained 5 µl preamplification product, 10 µl TaqMan Gene Expression Master Mix (#4369016, Applied Biosystems), and 1x of each TaqMan probe (ThermoFisher). The following reaction conditions were employed according to the manufacturer's instructions: 50 °C for 2 min, 95 °C for 10 min, then 40 cycles of 95 °C for 15 sec and 60 °C for 1 min. The relative level of mRNA was evaluated with the ΔΔCt method, using *ACTB* as an endogenous control.

### Immunocytochemistry

Cells were permeabilized with 0.1% Triton X‐100 (#85 111, Thermo Scientific) for 20–30 min at room temperature, then rinsed three times with D‐PBS (+/+). Following this, the cells were incubated for at least 1 h at room temperature in 1.5% BSA blocking buffer. After blocking, cells were incubated with the appropriate dilution of the desired primary antibodies overnight at 4 °C. To stain for stress granules, G3BP1 primary antibody (#ab109012, Abcam) was used at 1 µg mL^−1^, washed three times with D‐PBS (+/+), and incubated with secondary antibody AlexaFluor555 anti‐rabbit (#A21428, Thermo Fisher) at a 1:1000 dilution in blocking solution for 1 h at room temperature. To stain for nucleoli, the Nucleolus Bright Green dye (#NBP1‐86651, Novus Biologicals) was diluted in D‐PBS(+/+) at 1:1000. Post‐staining, the cells were rinsed three more times with D‐PBS (+/+) and stained with Hoechst 33342 (#H3570, Thermo Fisher) before imaging. All 96‐well plates generated were imaged using the Opera Phenix High‐Content Confocal microscope at a 40X magnification. Images of 16–20 unique frames per well (technical replicate, n = 3) for each biological replicate (N = 3) were acquired for quantification. Image analysis and quantifications were performed with the Harmony High‐Content Imaging and Analysis Software (Perkin Elmer). G3BP1 fluorescence was used to segment and quantify stress granule area, while Nucleolus Bright Green dye staining for nucleoli RNA was used to segment and quantify nucleoli area.

### Statistical Analysis

Statistical analyses, number of replicates, and p‐values for each experiment are indicated in the figures and/or their descriptions. Data acquisition and processing are described within the relevant sections of the Experimental Section. Statistical analyses were run in R.

## Conflict of Interest

Frank Pun and Alex Zhavoronkov are affiliated with Insilico Medicine, a commercial company developing and using generative artificial intelligence and other next‐generation AI technologies and robotics for drug discovery, drug development, and aging research. Insilico Medicine has developed a portfolio of multiple therapeutic programs targeting fibrotic diseases, cancer, immunological diseases, and age‐related diseases, utilizing its generative AI platform and a range of deep aging clocks and AI life models. The other authors declare no conflict of interest.

## Author Contributions

C.M.L., R.I.M., and M.V. conceptualized the study. C.M.L. coordinated the study and carried out data analysis. C.M.L., Y.B., and A.G.D. performed the experiments. All authors contributed to the writing, review, and editing of the manuscript.

## Supporting information



Supporting Information

Supporting Information

Supporting Information

Supporting Information

## Data Availability

The authors declare that all data supporting the findings of this study are available within this article.
