# Supplementary material for: Organization of the Proteostasis Network of Membraneless Organelles
Source: Adv Sci (Weinh). 2025 Jun 11;12(33):e00233. doi: 10.1002/advs.202500233 (PMC12412463; doi:10.1002/advs.202500233)
Supplement: Supplementary file 1 — Supporting Information [file ADVS-12-e00233-s001.pdf]

ADVANCED SCIENCE
Open Access

Supporting Information

for *Adv. Sci.*, DOI 10.1002/advs.202500233

Organization of the Proteostasis Network of Membraneless Organelles

Christine M. Lim, Yuqi Bian, Alicia González Díaz, Frank Pun, Alex Zhavoronkov, Richard I. Morimoto and Michele Vendruscolo**

SUPPLEMENTARY INFORMATION

Organization of the Proteostasis Network of Membraneless Organelles

Christine M. Lim[1], Yuqi Bian[1], Alicia González Díaz[1], Frank Pun[2], Alex Zhavoronkov[2], Richard I. Morimoto[3*] and Michele Vendruscolo[1*]

[1] *Centre for Misfolding Diseases, Yusuf Hamied Department of Chemistry, University of Cambridge, Cambridge CB2 1EW, UK*
[2] *Insilico Medicine, Hong Kong Science and Technology Park, Hong Kong, China*
[3] *Department of Biochemistry, Molecular Biology and Cell Biology, Rice Institute for Biomedical Research, Northwestern University, Evanston, IL 60208-3500, USA*

[*] Corresponding authors: mv245@cam.ac.uk

Supplementary Data 1. Benchmarking Protein-MLO localisation across 4 databases. Proteins and their reported MLO-localisation from 4 databases: DrLLPS [1], PhaSepDB [2-3], CD-CODE [4], and PhaSePro [5] were collated and compared. Only data from '*Homo sapiens*' were included from all 4 databases. MLO names across the databases were standardized and presented in column 2 'MLO'. PhaSePro was omitted from the quantification of the number of unique components per MLO across databases.

Supplementary Data 2. PN of MLOs. The PN of MLOs is made up of over 220,000 pairwise interactions. Each interaction involves one protein in the PN (column A) and one protein reported as present in one of 7 well-characterized MLOs (PML nuclear bodies, centrosomes, stress granules, P-bodies, nucleoli, post-synaptic densities, and nuclear speckles) (column C).

Supplementary Data 3. Lists of the centrality scores (degree and betweenness) for all proteins in their MLO regulatory networks, for each MLO.

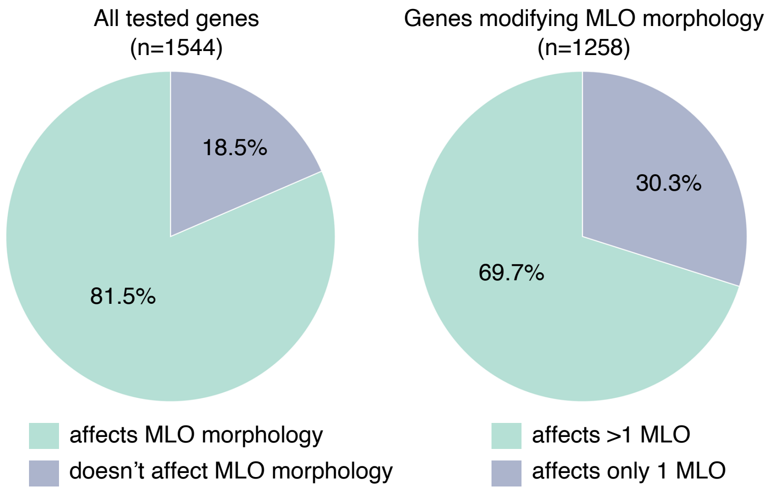

Figure S1. Specificity of genes previously found to affect the morphology of MLOs. In a large-scale study, a large majority (over 80%) of the of 1544 genes tested via siRNA knockdown were found to cause changes in the morphology MLOs [6]. In addition, about 70% of these genes affected the morphology of more than MLO, suggesting low specificity in the regulation of MLOs.

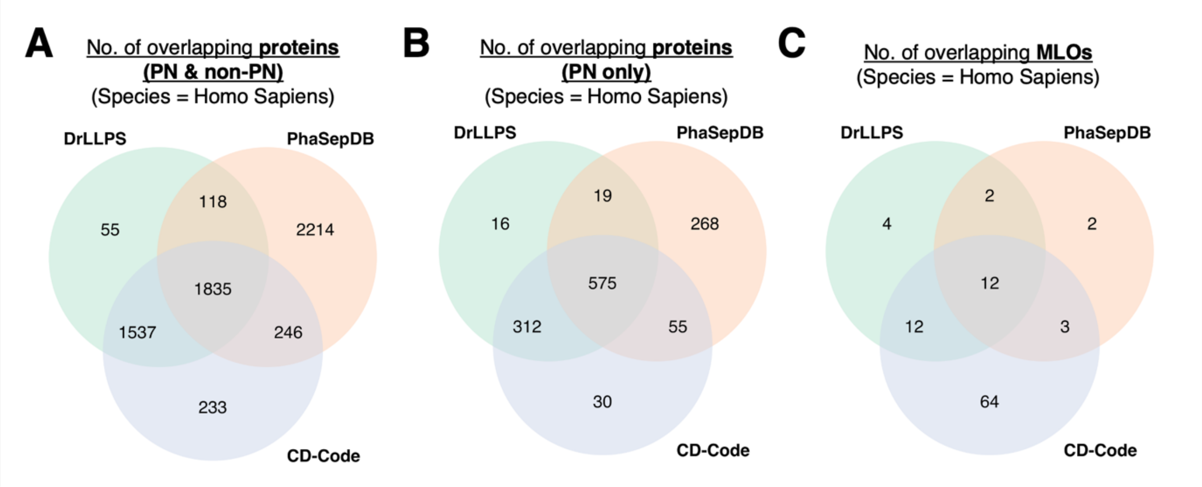

Figure S2. Comparison of DrLLPS, PhaSepDB, and CD-Code. Only data related to *Homo sapiens* were collated and compared across all 3 protein-MLO localisation datasets. **(A)** 1835 proteins (all proteins regardless of proteostasis involvement) are part of all 3 datasets; 1537 proteins are common between DrLLPS and CD-CODE; 246 proteins are common to CD-Code and PhaSepDB; 118 proteins are in both DrLLPS and PhaSepDB. Of the 3 datasets, DrLLPS has the least number of proteins (55 proteins) not reported by other datasets. **(B)** 575 proteins (proteins involved in proteostasis) are part of all 3 datasets; 312 PN proteins are common between DrLLPS and CD-CODE; 55 PN proteins are common to CD-Code and PhaSepDB; 19 PN proteins are in both DrLLPS and PhaSepDB. Of the 3 datasets, DrLLPS has the least number of PN proteins (16 PN proteins) not reported by other datasets. **(C)** Of all the MLOs profiled across 3 datasets, 12 MLOs were profiled by all DrLLPS, PhaSepDB, and CD-Code. The 12 MLOs are: Nuclear Speckles, Nucleoli, Paraspeckles, Stress Granules, Sam68 Nuclear Bodies, Centrosomes, Cajal Bodies, PML Nuclear Bodies, Nuclear Pore Complexes, Histone Locus Bodies, Nuclear Stress Bodies. Postsynaptic densities (PSDs) are annotated in both DrLLPS and CD-Code, but PSD components are reported together with components of presynaptic clusters in PhaSepDB.

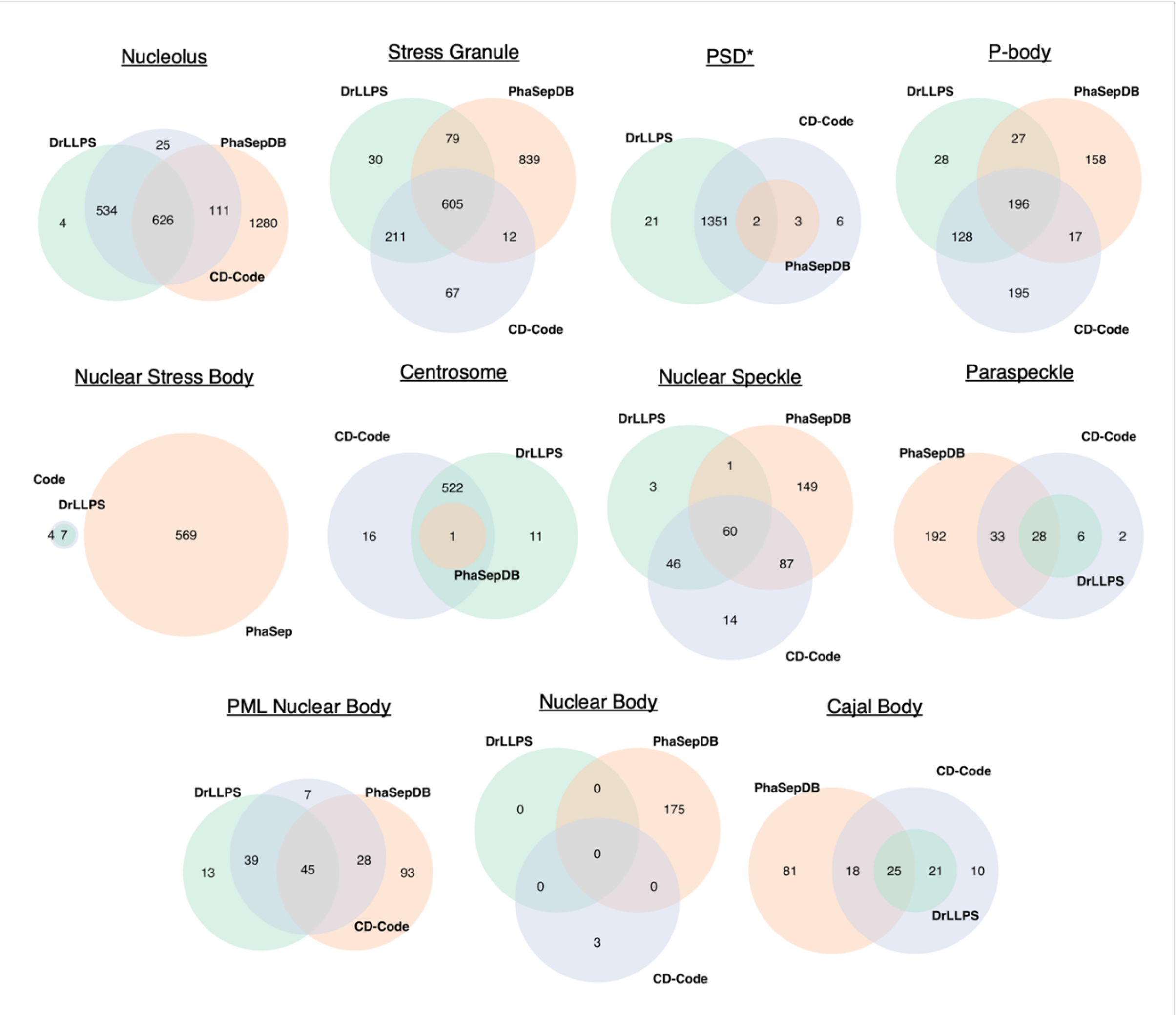

Figure S3. Consistency of protein-MLO localisation across DrLLPS, PhaSepDB, and CD-Code. 11 MLOs with more than 100 proteins (unique UniProt identifiers reported in at least 1 of 3 databases) were compared. Of the 11 MLOs compared, 7 show high consistencies in the reported protein-MLO localisation (at least 100 proteins that were common to at least 2 datasets). The 7 MLOs with high consistencies ('best-profiled') are the: Nucleolus, Stress Granule, Post Synaptic Density (PSD), P-body, Centrosome, Nuclear Speckle, and PML Nuclear Body. *components of the PSD in DrLLPS/CD-Code were combined with components of both the PSD and Presynaptic clusters (combined) in PhaSepDB.

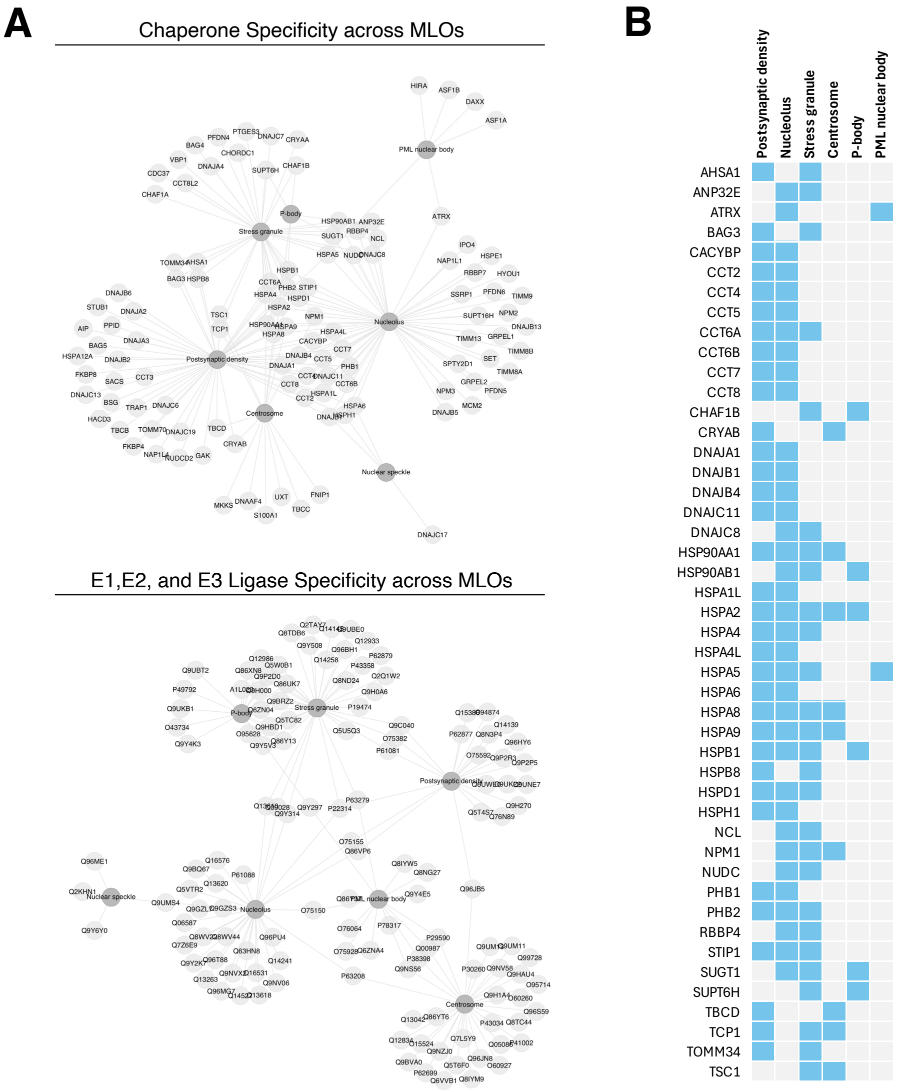

Figure S4. Molecular chaperones tend to be MLO promiscuous, while UPS E1, E2, and E3 ligases tend to be MLO specific. (A) Localisation patterns of molecular chaperones and E1, E2, and E3 ligases across MLOs. A large number of molecular chaperones (approximately 40 out of 220) are found to be shared across multiple MLOs. In contrast, E1, E2, and E3 ligases tend to be more specific to MLOs with few ligases common to multiple MLOs. **(B)** Localisation of promiscuous chaperones across MLOs. We note that in addition to the HSP70 family of proteins and their co-chaperones, several members of the small HSP family such as HSPB1 and HSPB8 appear to be promiscuous across multiple MLOs. Previous work has reported the role of HSPB8 in maintaining the dynamic of stress granules [7-8], as well as the role of HSPB1 in both regulating the dynamics of stress granule and TDP-43 containing condensates [7, 9-10]. In this work we focus on the hierarchial protein families such as HSP70 and their co-chaperones, and E1/E2/E3 ligases known to have a funneling effect on substrate specificity at lower levels.

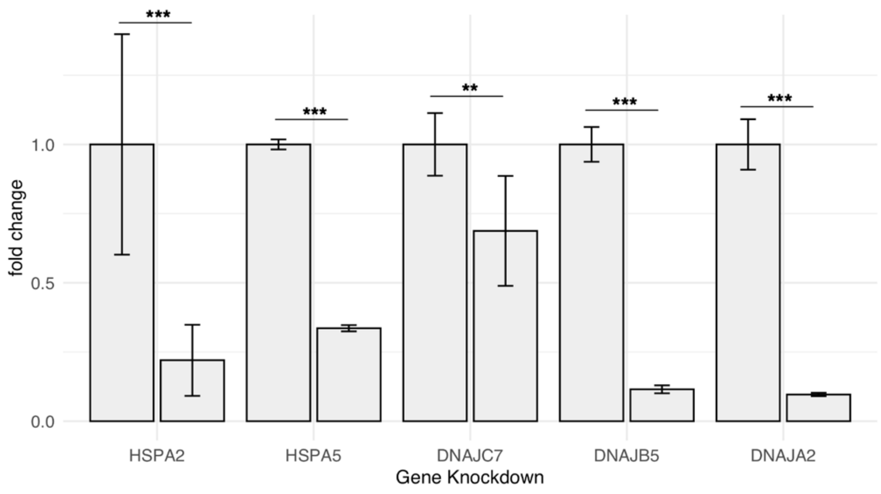

Figure S5. Validation of siRNA knockdown efficiency. PCR was carried out to validate the knockdown efficiency of all siRNAs used. The statistical significance was determined using the ANOVA test. (***) p-value < 0.01, and (**) p-value < 0.05.

Supplementary References

[1] W. Ning, Y. Guo, S. Lin, B. Mei, Y. Wu, P. Jiang, X. Tan, W. Zhang, G. Chen, D. Peng, *Nucl. Acids Res.* **2020**, *48* (D1), D288.

[2] C. Hou, X. Wang, H. Xie, T. Chen, P. Zhu, X. Xu, K. You, T. Li, *Nucl. Acids Res.* **2023**, *51* (D1), D460.

[3] K. You, Q. Huang, C. Yu, B. Shen, C. Sevilla, M. Shi, H. Hermjakob, Y. Chen, T. Li, *Nucl. Acids Res.* **2020**, *48* (D1), D354.

[4] N. Rostam, S. Ghosh, C. F. W. Chow, A. Hadarovich, C. Landerer, R. Ghosh, H. Moon, L. Hersemann, D. M. Mitrea, I. A. Klein, *Nature methods* **2023**, *20* (5), 673.

[5] B. Mészáros, G. Erdős, B. Szabó, É. Schád, Á. Tantos, R. Abukhairan, T. Horváth, N. Murvai, O. P. Kovács, M. Kovács, *Nucl. Acids Res.* **2020**, *48* (D1), D360.

[6] D. Berchtold, N. Battich, L. Pelkmans, *Mol. Cell* **2018**, *72* (6), 1035.

[7] M. Ganassi, D. Mateju, I. Bigi, L. Mediani, I. Poser, H. O. Lee, S. J. Seguin, F. F. Morelli, J. Vinet, G. Leo, *Mol. Cell* **2016**, *63* (5), 796.

[8] E. E. Boczek, J. Fürsch, M. L. Niedermeier, L. Jawerth, M. Jahnel, M. Ruer-Gruß, K.-M. Kammer, P. Heid, L. Mediani, J. Wang, *eLife* **2021**, *10*, e69377.

[9] S. Lu, J. Hu, O. A. Arogundade, A. Goginashvili, S. Vazquez-Sanchez, J. K. Diedrich, J. Gu, J. Blum, S. Oung, Q. Ye, *Nat. Cell Biol.* **2022**, *24* (9), 1378.

[10] X. Yan, D. Kuster, P. Mohanty, J. Nijssen, K. Pombo-García, A. Rizuan, T. M. Franzmann, A. Sergeeva, P. M. Passos, L. George, *bioRxiv* **2024**.
